# Essential oil and aromatic plants as feed additives in non-ruminant nutrition: a review

**DOI:** 10.1186/s40104-015-0004-5

**Published:** 2015-02-24

**Authors:** Zhaikai Zeng, Sai Zhang, Hongliang Wang, Xiangshu Piao

**Affiliations:** State Key Laboratory of Animal Nutrition, Ministry of Agriculture Feed Industry Centre, China Agricultural University, Beijing, 100193 China

**Keywords:** Antimicrobial, Antioxidant, Essential oils, Feed additives, Growth promoter, Gut function, Immunity

## Abstract

This paper summarizes the current knowledge regarding the possible modes of action and nutritional factors involved in the use of essential oils (EOs) for swine and poultry. EOs have recently attracted increased interest as feed additives to be fed to swine and poultry, possibly replacing the use of antibiotic growth promoters which have been prohibited in the European Union since 2006. In general, EOs enhance the production of digestive secretions and nutrient absorption, reduce pathogenic stress in the gut, exert antioxidant properties and reinforce the animal’s immune status, which help to explain the enhanced performance observed in swine and poultry. However, the mechanisms involved in causing this growth promotion are far from being elucidated, since data on the complex gut ecosystem, gut function, *in vivo* oxidative status and immune system are still lacking. In addition, limited information is available regarding the interaction between EOs and feed ingredients or other feed additives (especially pro- or prebiotics and organic acids). This knowledge may help feed formulators to better utilize EOs when they formulate diets for poultry and swine.

## Introduction

Antibiotics fed at sub-therapeutic levels have been widely utilized in the swine and poultry industries to improve growth rate and efficiency of feed utilization, as well as reduce morbidity and mortality [1]. However, many countries have restricted or even banned (i.e. the European Union) the use of antibiotics as feed additives due to increased concerns regarding the transmission and the proliferation of resistant bacteria via the food chain. The restriction on the use of antibiotics as feed additives has driven nutritionists and feed manufacturers to develop alternatives such as organic acids, feed enzymes, and pro- or pre-biotics. These substances are well established in animal nutrition. In contrast, plant extracts, especially EOs, are a new class of feed additives and knowledge regarding their modes of action and aspects of application are still rather rudimentary [2].

In recent years, EOs have attracted increased attention from the swine and poultry industries. However, they are not simple compounds, rather a mixture of various compounds (mainly terpenes and terpene derivatives) [3], which are concentrated hydrophobic liquids containing volatile aromatic compounds obtained from plants [4]. In terms of biological activity and effects, each individual chemical constituent has its own characteristic properties. This means that EOs are of a complex character with rather diverse effects. Furthermore, factors such as species, ecological factors and climatic conditions, harvest time, part of plant used and method of isolation all affect the chemical composition of EOs [4]. This variability complicates the assessment and application of EOs. The purpose of this paper is to provide an overview of the published data on the general applications of EOs in swine and poultry and discuss possible modes of action based on an *in vivo* model.

### Performance response generated by EOs

Numerous studies have documented the benefits of EOs on the performance of swine and poultry. Franz et al. [5] reviewed 8 reports with piglets and Windisch et al. [2] reviewed 11 reports with poultry. They reported that the average improvement in weight gain, feed intake and feed conversion induced by EOs were 2.0, 0.9 and 3.0% for piglets and 0.5, −1.6 and −2.6% for poultry, respectively. We collected data missed in the 2 reviews, as well as recently published data. For piglets, the improvement in performance was on average 10 and 3% while in poultry the improvement in performance was 3 and 3% for weight gain and feed conversion, respectively (Table 1). The different results for the two species are possibly caused by the different digestive physiology, the origin of the EOs or herb species, the quantity added to the feed and the environmental conditions used in the trial.

**Table 1:** **Effects of essential oils and aromatic plants on the performance of swine and poultry**

**Feed additive**	**Dose,mg/kg**	**Major components**	**Treatment effects (%, difference to control)**				**References**
			**Species**	**ADG**	**ADFI**	**FCR**	
Plant extract	150	5% Carvacrol (*Origanum spp.*), 3% cinnamaldehyde and 2% capsicum oleoresin	Weaned pigs	−5	−6	1	Manzanilla et al. [6]
	300			−2	-	−2	
Herbal extracts	7,500	Cinnamon, thyme, oregano and a carrier	Weaned pigs	−10	−17	8	Namkung et al. [7]
EO blend	300	Fenugreek (40%), clove (12.5%), cinnamon (7.5%) and carrier (40%)	Weaned pigs	7	5	−2	Cho, et al. [8]
Phytobiotics	1,000	Anis oil, citrus oil, oregano oil, and natural flavors	Nursery pigs	4	1	−2	Kommera et al. [9]
Plant extract	300	5% (wt/wt) Carvacrol, 3% cinnamaldehyde, and 2% capsicum oleoresin	Weaned pigs	33	26	−4	Manzanilla et al. [10]
Plant extract	300	5% (wt/wt) Carvacrol (*Origanum* spp.), 3% cinnamaldehyde (*Cinnamonum* spp.), and 2% capsicum oleoresin (*Capsicum annum*)	Weaned pigs	33	26	−4	Nofrarías, et al. [11]
Fennel	100	Fennel and caraway oil were obtained by steam distillation from fennel or caraway seeds	Weaned pigs	6	3	−3	Schone et al. [12]
Caraway	100			0	−1	−2	
EO blend	100	Buckwheat, thyme, curcuma, black pepper and ginger	Weaned pigs	0	-3	−4	Yan et al. [13]
EO blend	1,000	*Cinnamomum verum, Origanum vulgare spp., Syzygium aromaticum, Thymus vulgaris* and *Rosmarinus*	Weaned pigs	2	-	−2	Huang et al. [14]
EO blend	300	4.44 g of anise oil, 1.30 g of clove oil, and 2.0 g of cinnamon oil/kg of additive	Weaned pigs	10	5	−4	Maenner et al. [15]
EO blend	300	27.8 g of anise (*Pimpinella anisum)* oil, 12.5 g of clove (*Syzygium aromaticum*) oil, and 46.0 g of peppermint (*M. arvensis*) oil/kg of additive		7	4	−3	
EO blend	50	Thymol, cinnamaldehyde	Weaned pigs	11	7	−3	Li et al. [16]
	100			22	19	−2	
	150			22	15	−5	
EO blend	1,000	Oregano, which contained 60% active substance (Cymene, Terpinene, Carvacrol) and 40% carrier (dextrin)	Weaned pigs	2	2	−1	Zhang et al. [17]
Chinese medicinal herbs	1,000	*2*0% of each of *Dioscoreaceae batatas, A. macrocephala, G. uralensis* and *Platycodon grandiflorum*	Weaned pigs	16	-	−14	Huang et al. [18]
	3,000			13	-	−11	
EO blend	100	18% thymol and cinnamaldehyde (EOD)	Weaned pigs	12	1	−10	Li et al. [19]
EO blend	100		Weaned pigs	10	−1	−10	Zeng et al. [20]
Oregano	500		Finisher pigs	−10	−8	3	Janz et al. [21]
EO blend	100	Thyme, rosemary, oreganum extracts and kaolin	Finisher pig	4	−1	−5	Yan et al. [22]
	100			4	2	−2	
EO blend	25	Blend of EO containing 2.9% active ingredients including thymol	Broiler	5	4	−1	Jang et al. [23]
	50			3	5	1	
EO blend	100	*Syzigium aromathicum* (clove); *Cinnamon ceylanensis*; *Cinnamon camphocamphora* (cinnamon)	Broiler	−1		2	Isabel and Santos [24]
Oregano EO	250	Carvacrol 84.0%; thymol 1.8%	Broiler	3	4	0	Basmacioglu et al. [25]
	500			3	−3	−8	
Oregano EO	300	77.3% carvacrol, 9.6% thymol	Broiler	−7	−4	2	Kirkpinar et al. [26]
Garlic EO	300	2-propenyl thioacetonitril 43.2%, trisulfide methyl 2-propenyl 23.4%, disulfide di-2-propenyl 20.9%		−3	−4	0	
Oregano EO + garlic EO	150/150	Carvacrol 38.7%, thymol 4.8%, 2-propenyl thioacetonitril 21.6%, trisulfide methyl 2-propenyl 11.7%, disulfide di-2-propenyl 10.4%		−4	−5	−2	
EO blend	100	Cinnamaldehyde and thymol	Broiler	5	1	−3	Amerah et al. [27]
	100			2	2	0	
Thyme EO	1,000	Thymol 44.1%, p-cymene 32.0%, terpineol 9.6%, linalol 4.6%	Broiler	−4	−3	0	Cross et al. [28]
Oregano EO	300	Carvacrol 86.7%; thymol 3.3%; p-cymene 1.3%; γ-terpinene 1.3%	Broiler	3	2	−1	Roofchaee et al. [29]
	600			5	0	−5	
	1,200			3	−2	−4	
EO blend	125	Oregano, anis and citrus peel-active component (carvacrol)	Broiler	5	−2	−6	Hong et al. [30]
EO blend	150	Carvacrol, thymol, eucalyptol, lemon	Broiler	7	-	−3	Alali et al. [31]
	250			8	-	−5	
	500			15	-	−7	
EO blend	100	Basil, caraway, laurel, lemon, oregano, sage, tea, thyme	Broiler	7	0	−6	Khattak et al. [32]
	200			7	0	−7	
	300			6	−2	−6	
	400			6	0	−5	
	500			7	−2	−8	
Ginger EO	75	Zingiberene 27.2%; β-Sesquiphellandrene 13.7; Sabinene 13.4%; Ar-curcumene 10.7%; β-Bisabolene 9.9%;	Broiler	7	6	0	Habibi et al. [33]
	150			5	6	0	
Rosewood EO	150	Linalool 84.8%; Minor *oxigenated sesquiterpenes* 3.4%; α-terpineol 2.9%; geraniol 1.0%	Broiler	2	1	−1	Aguilar et al. [34]
	300			2	2	0	
	450			1	−1	−2	
	600			1	2	0	
Thymol	30	Thymol	Turkey	0	-	−1	Ginnenas et al. [35]
EO blend	30	10% thymol, 0.5% eugenol, 0.05% piperine		7	-	−8	

Another important consideration is the stability of EOs during feed processing. Maenner et al. [15] reported a considerable loss of activity of EOs when a pelleting temperature of 58°C was applied. These figures are smaller compared with conventional in-feed antibiotics, where advantages of 16.9% in weight gain (piglets) are reported in the literature [1]. However, in a recent feeding trial, Li et al. [19] compared the performance of piglets fed an unsupplemented control diet with that of piglets fed a diet supplemented with antibiotics or a combination of thymol and cinnamaldehye (Table 2). Weight gain, feed conversion and fecal consistency of pigs fed EOs was essentially equal to that of pigs fed antibiotics.

**Table 2:** **Effect of dietary essential oil and antibiotics on the performance and fecal consistency of weanling pigs** ^**1**^

**Item**	**Control**	**Antibiotic** ^**1**^	**Essential oil**	**SEM**	***P***
Phase 1 (d 0 to 7)					
Weight gain, g/d	354	378	416	28	0.33
Feed intake, g/d	473	478	502	26	0.71
Feed conversion	1.36	1.3	1.24	0.08	0.59
Phase 2 (d 8 to 35)					
Weight gain, g/d	465^b^	539^a^	513^a^	15	<0.01
Feed intake, g/d	860	937	861	26	0.07
Feed conversion	1.87	1.73	1.69	0.07	0.18
Overall (d 0 to 35)					
Weight gain, g/d	442^b^	505^a^	493^a^	15	0.02
Feed intake, g/d	783	846	789	24	0.13
Feed conversion	1.79	1.67	1.62	0.06	0.20
Feed consistency	1.53^b^	1.22^a^	1.30^a^	0.06	0.02

Li et al. [19].

^1^Control = Basal diet; Antibiotic = Basal diet supplemented with 150 mg/kg chlortetracycline, 80 mg/kg colistin sulfate, and 50 mg/kg kitasamycin); EO = Basal diet supplemented with 18 mg/kg of thymol and cinnamaldehyde.

^a-b^Means in the same row with different superscripts are significantly different (*P* < 0.05).

Aromatic herbs and EOs are often claimed to improve the flavor and palatability of feed, thus increasing voluntary feed intake resulting in improved weight gain. However, in a choice feed experiment conducted in growing pigs by Schöne et al. [12], the classification of fennel and caraway oils as flavor additives or as ‘appetite promoters’ in diets for pigs was questioned. Unfortunately, only 12 castrated male pigs (28 ± 1 kg) were used with 3 treatments and only a 4 day trial duration, which is weak due to the low level of replication and short feeding period used. Pigs may need a few days to adapt to the special flavor of EOs. Further studies are expected in this field to justify the assumption that herbs, spices and their extracts improve feed intake in pigs.

The application of EOs and aromatic plants in grower-finisher pigs seems unsuccessful. Janz et al. [21] and Yan et al. [22] failed to observe any improvement in performance generated by EOs or aromatic plants in finisher pigs. However, supplementation of EOs in sow diets, especially in lactation sow diets, has been attracting increasing interest. Miller et al. [36] reported that supplementation with 2 g/kg of a blend of EOs (Biomin P. E. P.), from 10 days prior to the estimated farrowing date through to weaning, improved the early lactation feed intake of sows, decreased sow weight loss during the first week of lactation and enhanced piglet body weight at weaning. In a study involving 2100 sows, Allan and Bilkei [37] reported that sows fed diets containing 1 g/kg oregano had higher voluntary feed intake, lower annual mortality rate (4.0 vs. 6.9%), reduced sow culling rate during lactation (8 vs. 14%), increased farrowing rate (77.0 vs. 69.9%), increased number of live born piglets per litter (10.49 vs. 9.95) and decreased stillbirth rate (0.91 vs. 0.81). Similar benefits generated by the feeding of EOs to sows have been reported by other authors [38-40].

### Regulation of gut microflora

EOs and aromatic plants are well known to exert antibacterial, antifungal and antiviral activity in *in vitro* experiments [2]. It is generally accepted that EOs are slightly more active against gram-positive than gram-negative bacteria [41,42]. The EO showed dose-dependent effects on cell integrity, as measured using propidium iodide, of Gram-positive bacteria. However, growth inhibition of Gram-negative bacteria, in contrast, occurred mostly without cell integrity loss [43]. Comparable *in vivo* studies also found inhibiting effects against pathogens such as *C. perfringens*, *E. coli* or *Eimeria* species (Table 3). The controlled pathogen load also contributed to healthy microbial metabolites, improved intestinal integrity and protection against enteric disease [44-47].

**Table 3:** **Effects of essential oils and aromatic plants on the microflora in swine and poultry**

**Feed additive**	**Dose, g/kg**	**Species**	**Measured responses**	**References**
Herbal extracts	7,500	Weaned pigs	Reduced *coliform* bacteria counts in fecal; less diverse of microbiota in ileal digesta base on PCR-DGGE	Namkung et al. [7]
EO blend	50-150	Weaned pigs	Increased *Lactobacillus* and decreased *E. coli* counts in feces	Li et al. [16]
EO blend	1,000	Weaned pigs	Increased *Lactobacillus* counts	Zhang et al. [17]
Chinese medicinal herbs	1,000/3,000	Weaned pigs	Increased *Lactobacilli* counts in ileum and decreased *Coliform* counts in colon	Huang et al. [18]
EO blend	100	Weaned pigs	Reduced *E. coli* and total aerobic bacteria in the rectum; increased *Lactobacilli* to *E. coli* ratio in colon	Li et al. [19]
Phytogenic additive	50-150	Weaned pigs	Microbial counts in feces (aerobes, gram negatives, anaerobes and lactobacilli) didn’t change	Muhl and Liebert [48]
EO blend	300	Broiler	Decreased intestinal *Clostridium*, but no effect on total organisms, *Streptococcus, Lactobacillus* and *Coliforms*	Kirkpinar et al. [26]
EO	100	Broiler	Increase in the mean numbers of bacterial species in the ileal content	Amerah et al. [27]
EO blend	1,000	Broiler	No change in cecal and fecal *Coliforms, Lactobacillus*, *C. perfringens* and total anerobes	Cross et al. [28]
Oregano EO	300-1,200	Broiler	Decreased cecal *E.Coli* but no effect for 1200 ppm; no effect on cecal *Lactobacilli*	Roofchaee et al. [29]
EO	125	Broiler	No change in cecal total bacteria, *Lactobaccilli*, *Enterococci*, *Coliforms* or *Salmonellae* colonization.	Hong et al. [30]
EO blend	150-500	Broiler	Decreased crop *Salmonella* but no effect for 150 ppm; no effect on *cecal Salmonella*	Alali et al. [31]
Thymol/EO	30	Broiler	Increased cecal *Lactobaccilli* and decreased *Coliform* but no effect on crop and ileum	Ginnenas et al. [35]
Oregano EO	300	Broiler	Lower bloody diarrhea, lesion score and oocyst numbers compared to control (*E. tenella* challenge)	Ginnenas et al. [49]
Oregano	330	Broiler	Decreased *C. perfringens* counts in cecum	Waldenstedt et al. [50]
EO blend	100	Broiler	Reduction of *C. perfringens* concentration in the jejunum and colon	Mitsch et al. [51]
Plant extract	100	Broiler	Reduction of *E. coli*, *C. perfringens* and *fungi* and increase of *Lactobacillus*	Jamroz et al. [52]
Oregano EO	0.5-1.25	Broiler	Oregano EO exhibited a strong bactericidal effect against *Lactobacilli* at both doses tested	Horošová et al. [53]
EO blend	100	Broiler	Increased ileal *Lactobacillus* counts coupled with decreased *E.Coli* counts	Rahimi et al. [54]
EO	500	Broiler	Decreased cecal *Staphylococci*, *Lactobaccilli* and *Enterobacteriaceae*	Placha et al. [55]
EO blend	25/50	Broiler	Decreased ileo-cecal *E.Coli*, and no change in *Lactobacilli*	Jang et al. [56]

Attention should also be paid to the potential negative effects induced by EOs on healthy intestinal bacteria. Horošová et al. [53] reported that oregano EO exhibited a strong bactericidal effect against *Lactobacilli* isolated from fecal samples of chickens fed diets with oregano. In a vivo anti-bacteria study, Thapa et al. [43] found that the beneficial commensal *Faecalibacterium prausnitzii* was sensitive to EO at similar or even lower concentrations than the pathogens. In addition, Cross et al. [28] and Muhl and Liebert [48] reported that EOs had no effect on the microbial population and composition in the digestive tract or fecal excretions of broilers and pigs.

In a review, Brenes and Roura [41] contended that minor components are critical to the bacteriostatic activity of EOs and may have synergistic effects. For example, carvacrol and thymol, the two structurally similar major components of oregano essential oil, were found to give an additive effect when tested against *S. aureus* and *P. aeruginosa* [57]. Cymene, a biological precursor of carvacrol, was found to have a higher preference for liposomal membranes, thereby causing more expansion. By this mechanism cymene probably enables carvacrol to be more easily transported into the cell so that a synergistic effect is achieved when the two are used together [58]. However, the major components of EOs obtained from conifers were reported to be more bacteriostatic than the crude essential oil of fir and pine, but were less active or had similar activity as the EO of spruce for *L. monocytogenes* 4 b and ½ c [40,59,60]. Therefore, it is likely that the other components, or combinations of the different major components, have double-edged effects (negative or positive) on the antimicrobial activity of the EOs from fir and pine. These studies indicate that there is still much work to do in order to develop a blend of EOs with better antimicrobial properties.

### Impact on nutrient absorption and gut morphology

EOs have been documented to improve nutrient digestibility in swine [15,19,21,61] and poultry [25,62]. The improvement in nutrient absorption may be partly explained by increased secretions of saliva, bile and enhanced enzyme activity [56,63-65]. However, Muhl and Liebert [66] did not observe improved nutrient digestibility and enhanced pancreatic and duodenal activity of trypsin and amylase in weaned piglets fed diets containing a phytogenic product having carvacrol, thymol and tannins as key constituents. The inconsistent results in apparent digestibility may be caused by endogenous loss resulting from a stimulated secretion of mucus induced by plant extracts [67].

The improved nutrient absorption may allow appropriate modifications to diet nutrient density. In a randomized complete block design, Zeng et al. [20] investigated the acceptance of commercial EOs in low energy density weaned pig diets with wheat and extruded full-fat soybean as the major ingredients. The piglets could freely choose between a standard energy density diet (DE = 3,400 kcal/kg) or a low energy density diet (DE = 3,250 kcal/kg) with 0 or 0.25 g/kg EOs (4.5% cinnamaldehyde and 13.5% thymol). EO supplementation significantly increased weight gain and improved the apparent digestibility of dry matter, crude protein and energy compared with pigs fed the low energy density control diet. Supplementation of EOs to a low-energy pig diet has beneficial effects and leads to similar performance compared with a standard energy density diet (Table 4).

**Table 4:** **Effects of dietary essential oil on the performance, fecal consistency and nutrient digestibility of weaned pigs** ^**1**^

**Item**	**PC**	**NC**	**EO**	**SEM**
Performance				
Weight gain, g/d	382^a^	348^b^	383^a^	4.50
Feed intake, g/d	633	636	631	11.98
Feed conversion	1.65^a^	1.82^b^	1.64^a^	0.04
Feed consistency	1.42^b^	1.44^b^	1.29^a^	0.07
Nutrient digestibility, %				
Dry matter	81.2^a^	79.2^b^	81.2^a^	0.48
Crude protein	79.3^a^	73.3^b^	79.2^a^	0.85
Energy	79.9^a^	76.3^b^	81.1^a^	0.57
Calcium	56.3	57.0	59.5	1.65
Phosphorus	56.3	56.0	60.0	1.61

Zeng et al. [20].

^1^Values represent the mean of twelve pens with four pigs per pen. The dietary treatments were: PC (positive control); NC (negative control, 150 kJ/kg DE lower than the PC diet); EO (NC diet supplemented with 0.025% EO product which contained at least 4.5% cinnamaldehyde and 13.5% thymol).

^a,b^Means in the same row with different superscripts are significantly different (*P* < 0.05).

Decreased numbers of pathogenic bacteria in the gut may improve the ability of epithelial cells to regenerated villus and thus enhance intestinal absorptive capacity [68]. It is reasonable to expect such an effect by EOs due to their well-documented inhibitory effects against pathogens. However, the literature is equivocal regarding the use of EOs as feed additives in relation to gut morphology. There are reports that show increased, unchanged as well as reduced villus length and crypt depth in the jejunum and colon for broilers and piglets fed EOs [6,10,19,20,52,69]. Considering the different reactions in gut morphology, Windusch et al. [70] hypothesized that one aspect of the phytogenic action of EOs seems to be irritation of intestinal tissues leading to reduced intestinal surface. In contrast, beneficial effects on gut health (i.e. reduced pathogen pressure) could favor increased villus length and gut surface. Consequently, the overall impact of EOs on gut morphology seems to depend on the balance between tissue irritation and beneficial effects on intestinal hygiene.

### Immune status

The gastrointestinal tract’s immune system is often referred to as gut-associated lymphoid tissue (GALT), which possesses the largest mass of lymphoid tissue and plays an important role in antigen defense in the human body [71]. In the results presented by Kroismayr et al. [72], using the techniques of quantitative real time-PCR and gut tissue morphology, EO and avilamycin significantly decreased the expression of the transcriptional factor NFκB, the apoptotic marker TNFα and the size of Peyer’s patches in the intestine of weaned piglets, as well as the proliferation marker cyclin D1 in the colon, mesenteric lymph nodes and spleen. Reduced numbers of intraepithelial lymphocytes in the jejunum and reduced B lymphocytes in mesenteric lymph nodes were also observed by Manzanilla et al. [10,69] and Nofrairas et al. [11]. This might serve as direct evidence for a lower need for immune defense activity in the gut due to the antimicrobial action of EOs. The relieved intestinal immune defense stress may partly contribute to nutrient allocation towards growth rather than immune defense.

Investigations conducted under practical conditions of large-scale animal production have shown better responses to EO treatment than more recent studies conducted under controlled experimental conditions with a higher level of hygiene [5]. This might be explained by a lower pathogen pressure in the intestine and an improved immune status. Supplementing EOs has been reported to improve the immune status of piglets after weaning, as indicated by an increase in lymphocyte proliferation rate, phagocytosis rate, as well as in IgG, IgA, IgM, C3 and C4 serum levels [16,19,20]. Walter et al. [73] reported that pigs fed a diet with 3 g/kg oregano (60 g carvacrol and 55 g thymol per kilogram) had higher proportions of CD4:CD8, MHC class II antigens, and non-T/non-B cells in peripheral blood lymphocytes compared with pigs fed a control diet.

The bioactive substances are quickly absorbed after oral, pulmonary, or dermal administration and most are metabolized and either eliminated by the kidneys in the form of glucuronide or exhaled as CO_2_ [74]. The absorbed component might initiate an immune response indicated by changes in blood immunological parameters while the unabsorbed component may contribute to relief from intestinal immune defense stress. However, the precise mechanisms through which EOs function are not clear and further investigations are necessary.

### Anti-oxidative effects

Stability is very important to minced meat during further processing or after cooking, or as surface treatments for whole cuts prior to storage. In order to prolong the storage stability of foods, synthetic antioxidants are used for industrial processing. Nevertheless, the use of some of the common synthetic antioxidants such as butylated hydroxytoluene (BHT) and butylated hydroxyanisole (BHA) has come into question due to their suspected carcinogenic potential as evidenced by toxicologists [75]. In addition, a general consumer rejection of synthetic food additives has been observed in recent times. For these reasons, there is an increasing interest in studies involving natural additives for use as potential antioxidants.

Herbs of the Labiatae family, particularly rosemary, oregano and sage, have been extensively studied for their antioxidant activity [41]. The potential of dietary EOs and aromatic plants to improve the oxidative stability of meat obtained from broilers, hens or turkeys, has been demonstrated in a series of studies [76-83]. However, Simitzis et al. [84] and Janz et al. [21] reported that dietary oregano EO failed to improve the lipid oxidation status of pork. This may be explained by the different fatty acid composition in the meat of poultry and swine. Although poultry meat contains a low lipid content, its relative concentration of polyunsaturated fatty acids is higher (60 vs 17%, of total fat content) than pork [21,85]. Thus, poultry meat is particularly susceptible to oxidative deterioration, which might contribute to a robust response on the lipid oxidation status of poultry meat that was generated by dietary EOs supplementation.

Beside benefits on meat quality, EOs or plant extracts are also reported to improve redox balance in different organs [55,86], and attenuate oxidative injury induced by different physiological stressors [87-89]. Table 5 shows the results of an experiment where different concentrations of ginger root powder and its EOs were fed to broilers raised under heat stress conditions [33]. Broilers which received 150 mg/kg ginger EO had increased total superoxide dismutase (TSOD) activity and decreased malondialdehyde (MDA) concentrations in the liver compared with a control group. Dietary supplementation of vitamin E, ginger root powder or its EO, increased total antioxidant capacity (TAC) and decreased MDA concentrations in serum compared with a control group.

**Table 5:** **Effect of ginger herb and its essential oil on antioxidant parameters and malondialdehyde in the erythrocytes, serum and liver of broilers raised under heat stress** ^**1**^

**Item**	**Control**	**VE 100**	**H 7.5**	**H 15**	**EO 75**	**EO150**	**SEM**	***P***
Erythrocytes								
Glutathione Peroxidase, U/mg Hb	35	36.6	36.9	36	34.5	34.8	0.63	0.87
Superoxide dismutase, U/mg Hb	1,414	1,398	1,268	1,243	1,270	1,210	27.80	0.16
Catalase, K/mg Hb	0.7	0.4	0.9	0.6	0.7	0.7	0.08	0.63
Serum								
Total antioxidant capacity, mmol/L	0.8^b^	1.0^a^	1.0^a^	1.0^a^	0.9^a^	1.0^a^	0.02	0.01
Malondialdehyde, nmol/mL	3.2^a^	2.5^bc^	2.2^cd^	2.1^d^	2.7^b^	2.6^bc^	0.08	0.05
Liver								
Glutathione Peroxidase, U/mg protein	0.5	0.5	0.5	0.5	0.5	0.5	-	0.76
Superoxide dismutase, U/mg protein	3.6^b^	4.0^ab^	3.7^b^	4.0^ab^	4.3^ab^	4.8^a^	0.12	0.05
Catalase, K/mg protein	0.3	0.3	0.3	0.2	0.4	0.3	0.03	0.71
Malondialdehyde, nmol/mL protein	5.3^a^	4.4^ab^	3.3^bc^	2.2^c^	2.3^c^	2.5^c^	0.30	0.01

Habibi et al. [33].

^1^Values are the mean of 4 replicates. Control = Basal diet without supplementation; VE 100 = Basal diet plus 100 mg/kg vitamin E; H 7.5 or H 15 = Basal diet plus 7.5 or 15 g/kg of ginger root powder; EO 75 or EO 150 = Basal diet plus 75 or 150 mg/kg of ginger essential oil.

^a-d^Means in the same row with different superscripts are significantly different (*P* < 0.05).

### The efficacy of EOs

There is limited information concerning the interaction between EOs and nutritional factors (such as nutrient level, type of basal diet, as well as synergistic or antagonistic effects with other feed additives). Jamroz et al. [67] investigated the influence of diet type (corn vs. wheat and barley) on the ability of plant extracts (100 mg/kg containing 5% carvacrol, 3% cinnamaldehyde and 2% of capsicum oleoresinon) to modify morphological and histochemical characteristics of the stomach and jenunal walls in chickens. Their results showed significantly more jenunal wall villi in chickens fed the maize diet supplemented with plant extracts.

The incorporation of carvacrol, cinnamaldehyde, and capsicum oleoresin promotes positive and negative changes in digestive function, intestinal epithelium, microbial ecology, and fermentation in weaned pigs depending on the amount of protein included in the diet [69]. In a study conducted to investigate the effects of three doses of individual and combined dietary supplements of specific blends of organic acids and EOs on broiler performance, Bozkurt et al. [90] concluded that a combination of acidifiers and EOs may allow a reduced dosage to be used due to their synergistic effects.

## Conclusions

The search for alternatives to antibiotics has generated considerable interest in recent years. The new generation of feed additives includes herbs and essential oils, and their beneficial effects for animal production have been well documented [2].

Although most of the latest research has noted the major components and original sources of EOs *in vivo* trials, only a few papers have identified the quantity of the principle components present. In addition, Brenes and Roura [41] argued that minor components present are critical to the activity of EOs and may have a synergistic influence. Sometimes the minor components may counteract the exerted effects. Therefore, in the future, the detailed constituents of EOs are needed to be determined in order to assess their different biological effects. In this way, it may be possible to compare different EO products and formulate mixtures that optimize their efficacy.
