# Correction to: Essential oil and aromatic plants as feed additives in non-ruminant nutrition: a review

**DOI:** 10.1186/s40104-020-00467-w

**Published:** 2020-05-11

**Authors:** Zhikai Zeng, Sai Zhang, Hongliang Wang, Xiangshu Piao

**Affiliations:** State Key Laboratory of Animal Nutrition, Ministry of Agriculture Feed Industry Centre, China Agricultural University, Beijing, 100193 China

**Correction to: J Animal Sci Biotechnol 6, 7 (2015)**

**https://doi.org/10.1186/s40104-015-0004-5**

Following publication of the original article [1], the authors identified an error in the author name **Zhikai Zeng**.

The incorrect author name is: Zhaikai Zeng

The correct author name is: Zhikai Zeng
